# Baseline MMP expression in periapical granuloma and its relationship with periapical wound healing after surgical endodontic treatment

**DOI:** 10.1186/s12903-021-01904-6

**Published:** 2021-11-03

**Authors:** Muhammad Adeel Ahmed, Muhammad Faraz Anwar, Khalid Ahmed, Marziya Aftab, Fizza Nazim, Muhammad Furqan Bari, Mohammed Mustafa, Fahim Vohra, Ali Alrahlah, Nouman Mughal, Syed Hani Abidi

**Affiliations:** 1 Department of Operative Dentistry and Institute of Biomedical Sciences, Dow University of Health Sciences, Karachi, Pakistan; 2 Department of Restorative Dentistry and Endodontics, College of Dentistry, King Faisal University, Al-Ahsa, Saudi Arabia; 3 Department of Biological and Biomedical Sciences, Aga Khan University, Karachi, Pakistan; 4 Department of Biochemistry, Bahria University Medical and Dental College, Karachi, Pakistan; 5 Department of Operative Dentistry, Dr. Ishrat-ul-Ebad Khan Institute of Oral Health Sciences, Dow University of Health Sciences, Karachi, Pakistan; 6 Department of Pathology, Dr. Ishrat-ul-Ebad Khan Institute of Oral Health Sciences, Dow University of Health Sciences, Karachi, Pakistan; 7 Department of Conservative Dental Sciences, College of Dentistry, Prince Sattam Bin Abdulaziz University, Al-Kharj, Saudi Arabia; 8 Department of Prosthetic Dental Sciences, Engineer Abdullah Bugshan Research Chair for Dental and Oral Rehabilitation, College of Dentistry, King Saud University, Riyadh, Saudi Arabia; 9 Department of Restorative Dental Sciences, Engineer Abdullah Bugshan Research Chair for Dental and Oral Rehabilitation, College of Dentistry, King Saud University, Riyadh, Saudi Arabia; 10 Department of Surgery, Aga Khan University, Karachi, Pakistan

**Keywords:** MMP expression, Periapical granuloma, Periapical wound healing, Surgical endodontic treatment

## Abstract

**Background:**

Matrix metalloproteinases (MMPs) catalyzes the degradation of the extracellular matrix components and have a major role in many physiological processes including wound healing. In the current study, we examined the correlation of baseline MMPs 1, 2, 7, and 9 expressions with periapical wound healing after surgical endodontic treatment.

**Methods:**

27 patients aged between 15 and 57 years presenting with chronic apical periodontitis or chronic apical abscess of an anterior tooth with previously attempted or failed root canal treatment were included in this study. During surgical endodontic treatment, tissue from the periapical lesion sample was collected and used for gross histopathological analysis as well as mRNA expression analysis of MMPs 1, 2, 7, and 9. Patients were recalled for follow-up after 6  months to evaluate the healing status both clinically and radiographically and healing was correlated with baseline MMP expression.

**Results:**

Out of 27 patients, healing was observed in 15 patients at the end of 6 months, and in 21 patients after 12 months.. Six patients showed no healing even after 12 months. Analysis of baseline MMP 1, 2, 7, and 9 expression levels with healing status showed the mean relative expression of MMP2 and MMP9 to be considerably increased in the non-healing group as compared to the healing group.

**Conclusion:**

Overexpression of MMP2 and MMP9 may be considered as a potential prognostic biomarker for periapical wound healing after surgical endodontic treatment. However, further studies are desirable to establish its precise relationship with periapical wound healing.

## Introduction

Periapical lesions are characterized as inflammatory osteolytic lesions of peri-radicular tissues of the jaw having an endodontic origin. Periapical granuloma (PG), the most common type of periapical lesions, develops as a result of continuous antigenic stimulation from an infected root canal [1]. Radiographically, these lesions appear as a unilocular radiolucency surrounding the apex of the necrosed tooth. Histologically, periapical granuloma comprises granulation tissue having mixed inflammatory infiltrates of plasma cells, lymphocytes, macrophages, eosinophils, and few neutrophils; newly developed blood vessels and nerve fibers [2]. Upon reaching the periapical tissues through the apical foramina, bacterial toxins from necrosed pulp initiate a cascade of local inflammatory responses. As a result, several proinflammatory cytokines, chemokines, immunoregulatory mediators, and neuropeptides are released into the periapical tissue environment that causes degradation of the extracellular matrix (ECM) and periapical bone destruction [3].

Periapical granulomas are primarily treated by conventional endodontic treatment. However, surgical endodontic treatment is indicated in case of periapical lesions healing failure after conventional root canal treatment or appropriate treatment cannot be performed by non-surgical means. It has now become clinically evident that patients may respond differently to periapical wound healing even if the treatment is performed following the same protocol. It has been suggested that individual genotypes may be responsible for this difference in wound healing [3]. Furthermore, altered expression of certain markers in the milieu of the lesion are also attributed to the outcome of healing [3].

Wound healing is characterized by a series of events including inflammation, granulation tissue formation, angiogenesis, and tissue remodeling. These processes occur in all tissues irrespective of the type of injury [4]. Several studies have indicated the involvement of matrix metalloproteinases (MMPs) in the catabolic turnover of bony matrix and ECM components in the periapical region. MMPs are a group of zinc and the calcium-dependent endogenous membrane-bound enzyme that mediate the degradation and remodeling of ECM and bony matrix [5]. These enzymes are produced by a variety of cells including fibroblast, endothelial cells, epithelial cells, inflammatory cells, and cementoblast-like cells. To date, more than 25 MMPs are known to play vital roles in physiological processes like tissue morphogenesis, repair, remodeling, and wound healing. However, many studies have indicated the dual role of MMPs during periapical inflammation, where innate and adaptive immune responses are activated to combat the invading pathogens and their by-products from devitalized teeth [6, 7]. For example, Belmar et al*.*[8] found an increased concentration of MMP-2 and MMP-9 in the gingival crevicular fluid of patients having a periapical infection, while Ahmed et al*.* [9] reported that higher expression of MMP-9 in periapical granuloma suggested its active role in the progression of these lesions. Furthermore, Hadziabic et al*.* [10] found an increased level of MMP-1 and MMP-2 in periapical granuloma as compared to cyst although without evidence of statistical significance. While numerous studies have concluded that elevated levels of MMPs lead to chronic non-healing of wounds [9, 11, 12].

However, prospective clinical studies elucidating the role of baseline gene expression in periapical wound healing after surgical endodontic treatment have rarely been documented. This study was based on the hypothesis that there is a correlation between baseline MMPs 1, 2, 7, 9 expressions in periapical granuloma and periapical wound healing after surgical endodontic treatment. A prospective longitudinal clinical study to evaluate the baseline expression of MMPs, and its correlation with clinical and radiographical periapical wound healing after apicoectomy can highlight baseline MMP expression as a potential prognostic biomarker for periapical wound healing and may aid in the provision of patient-centered treatment modalities as an aid to surgical endodontic treatment.

## Material and methods

### Patient selection

Study participants were recruited from the Department of Operative Dentistry, Dow University of Health Sciences during November 2017 to October 2019. The study protocol was approved by Institutional Review Board, Dow University of Health Sciences (Ref: IRB-862/DUHS/Approval/2017/50). Informed consent was taken from all patients before initiating the treatment.

Participants aged between 15 and 57 years, presenting with chronic apical periodontitis or chronic apical abscess of an anterior tooth with previously attempted or failed root canal treatment, radiographically shown as radiolucency around the root apex (greater than 4 mm) with the loss of lamina dura, periodontal ligament space and patients with persistent periapical lesion developing in a single-rooted tooth that has encountered trauma before root closure were enrolled in this study. A total of 52 patients who met the inclusion criteria of the study were initially included in the study. These patients were followed for up to 6 months after conventional re-root canal treatment  and assessed for healing. In the end, out of 52, only samples from 27 participants were selected for genetic analysis because of various reasons, such as failure to meet inclusion criteria because of evidence of healing (disappearance of signs and symptoms), insufficient periapical tissue for nucleic acid extraction, and compromised tissue quality. Such approach and sample size have also been utilized in other studies [13–15]. Medically compromised patients with any uncontrolled systemic disease or ASA Level III, multi-rooted teeth, single-rooted teeth with less than 4 mm periapical lesion, and patients with pre-existing conditions such as periodontal disease, and pregnant or lactating women, patients in which the healing was observed after conventional re-root canal treatment were excluded from the study.

### Treatment protocol and tissue retrieval

A preoperative digital periapical radiograph was taken with cone indicator and reference marker, placed over the sensor. Conventional re-root canal treatment was performed, patients were recalled after 3 to 6 months for evaluation of periapical healing, those patients in which periapical healing was not observed were scheduled for periapical surgery. Local anesthesia 1:80,000 lidocaine with epinephrine was administered and a full-thickness mucoperiosteal flap was elevated. After identifying the periapical lesion site, a window was created by removing the cortical bone with a small round bur #2 (Mani, Japan) in a slow-speed handpiece. The periapical lesion was removed with the help of curettes (Hilbro, Japan). Apicoectomy and retrograde cavity preparation was performed with the help of ultrasonic tips (Pro ultra, DENTSPLY Maillefer, Switzerland) and the retrograde filling was done with MTA (Pro-root MTA, DENTSPLY Tulsa Dental Specialties, USA). The flap was then repositioned and sutured with a 3/0 silk suture (ETHICON, LLC).

Tissue from the periapical lesion was collected and divided immediately into two fragments: one fragment was transferred in RNA later buffer filled 1.5 mL sterilized centrifuge tubes and kept in − 80 °C until homogenized to extract RNA, the second fragment was used for histopathological analysis.

### Tissue processing for histopathological analysis

After removal from the root apex, the adhering diseased tissues were immediately stored in 10% buffered formalin until histopathological analysis. Each lesion tissue was then placed in a tissue processor (PT09, Lupetec, Sao Carlos, SP, Brazil) where they were treated with increasing concentration of ethanol, proceeded with xylol before being embedded in paraffin. Later these paraffin blocks were cut into 5-µm sections for histopathological examination. During deparaffinization, the 5-µm sections were treated with two sequences of xylol, hydrating in decreasing concentration of ethanol, and washed with tap and distilled water before placing it in hematoxylin for 3 min. The sections were then washed in tap water for 15 min followed by immersion into 70% ethanol and then keeping it in eosin for 5 min. Lastly, the samples were immersed in absolute ethanol four times and three times in xylol before being mounted by coverslip using Enthelan® (Merck, Darmstadt, Germany). Microscopic analysis of the hematoxylin–eosin-stained slides was performed using a Nikon Eclipse E200 optical microscope (Nikon Instruments Inc., Tokyo, Japan). Only the lesions classified as Periapical Granuloma were included in this study. The periapical granuloma was identified as granulation tissues surrounded by fibrous connective tissues showing the presence of macrophages, lymphocytes, plasma cells, giant cells, fibroblasts, and masts cells.

### Tissue processing for RNA extraction and cDNA synthesis

Total RNA was purified from each of the frozen periapical granuloma tissue using bead mill homogenization as per the manufacturer’s instructions. The tissue was weighed out in 2 mL centrifuged tube already containing 0.4 g of 0.5 mm glass beads and 1% β-mercaptoethanol containing 500µL of RLT Buffer (Qiagen, Hilden, Germany). The tube was then subjected to the bead mill homogenization process in Omni bead ruptor 24 (Omni International, Kennesaw, GA, USA). Total RNA was then purified from homogenate using RNeasy Mini kit (Qiagen, Hilden, Germany) according to the manufacturer’s instructions. RNA concentration and purity were quantified by nanodrop and aliquoted to save at − 80 °C until further use.

RNA was reverse transcribed by using an M-MLV reverse transcriptase kit (Promega, Madison, WI, USA). RNA template (5µL) was mixed with 1µL OligodT (0.5 µg/µL), 1µL dNTPs 10 µM and 8µL Nuclease free water and was incubated on the preheated block at 65 °C for 5 min. At the end of incubation, the reaction mixture was immediately chilled on ice for 5 min then briefly spin to bring the contents down to the bottom of the tube. This reaction was combined with a reaction mixture containing 4µL M-MLV RT 5 × reaction buffer and 1µL M-MLV Reverse transcriptase (10,000U) to make the volume up to 20µL. This reaction mixture was incubated at 50 °C for 30 min, 85 °C for 5 min, and 4 °C for hold in Eppendorf (Hamburg, Germany) thermal cycler.

### Quantitative polymerase chain reaction for metalloprotease genes

cDNA samples were used to perform quantitative-PCR (qPCR) to measure the expression levels of MMP -1, -2, -7, and -9 genes. β-actin served as a housekeeping gene and was also used to normalize the results in a qPCR using respective primer sets. A list of primers used to measure the levels of MMP, and β-actin is given in Table 1.

**Table 1:** Name of target genes and respective primer sets used to quantify mRNA levels in qPCR

Gene	Forward Primer (5ʹ to 3ʹ)	Reverse Primer (5ʹ to 3ʹ)
Β-actin	GCGCGGCTACAGCTTCA	CTCCTTAATGTCACGCACGAT
MMP1	AAAATTACACGCCAGATTTGCC	TGTTGGTCCACCTTTCATCTTC
MMP2	TACAGGATCATTGGCTACACACC	GGTCACATCGCTCCAGACT
MMP7	GAGTGAGCTACAGTGGGAACA	CTATGACGCGGGAGTTTAACAT
MMP9	TGTACCGCTATGGTTACACTCG	GGCAGGGACAGTTGCTTCT

For qPCR analysis, a 20uL reaction mixture was prepared using the following recipe: 2uL of cDNA, 0.6µL (10 pmol/µL) of forward and reverse primers, and 10uL of Evergreen qPCR master mix (ABM, Canada). The qPCR reaction was run on CFX96™ Real-Time PCR System (BIO-RAD, USA), using thermocycling protocol: 10 min at 95 °C, 40 cycles of 15 s at 95 °C and 30 s at 57 °C. Only four samples, which had low total RNA concentrations, were allowed to run up to 60 cycles. Melt curve (55–95 °C) analysis was performed at the end of 40/60 cycles to verify the identity of PCR products. All reactions were run in triplicate. The relative gene expression was calculated using the following formula:

$${\text{Relative}}\,\% \,{\text{gene}}\,{\text{expression}}:\quad {\text{A}}/{\text{B}} \times 100$$

where A = average Ct for housekeeping gene, B = average Ct for the gene of interest (MMPs).

In the next step, we applied ANOVA with Tukey’s multiple comparison test to determine the significant difference in relative gene expression between all tested MMPs in healer and non-healer, where *p* < 0.05 was considered as a statistically significant value.

## Results

### Patient profile and clinical features

The demographic data of the patients suggest that the mean age was 22.81 ± 7.45. Twenty male and seven female patients were included in this study. Out of 27 patients, 5 were smokers. The pre-operative clinical presentation of the patients is summarized in Table 2. More than half (55.55%) of the patients complained of either pain, swelling, and/or sinus tract. Tenderness to percussion was present in 20 (74.07%) patients.

**Table 2:** Clinical presentation of the study participants before periapical surgery

Clinical parameters	Sign/symptom	Frequency	Percentage
Pain/swelling and/or sinus tract	Present	15	55.55
	Absent	12	44.44
Tenderness to percussion	Present	20	74.07
	Absent	7	25.92

**Fig. 1:**
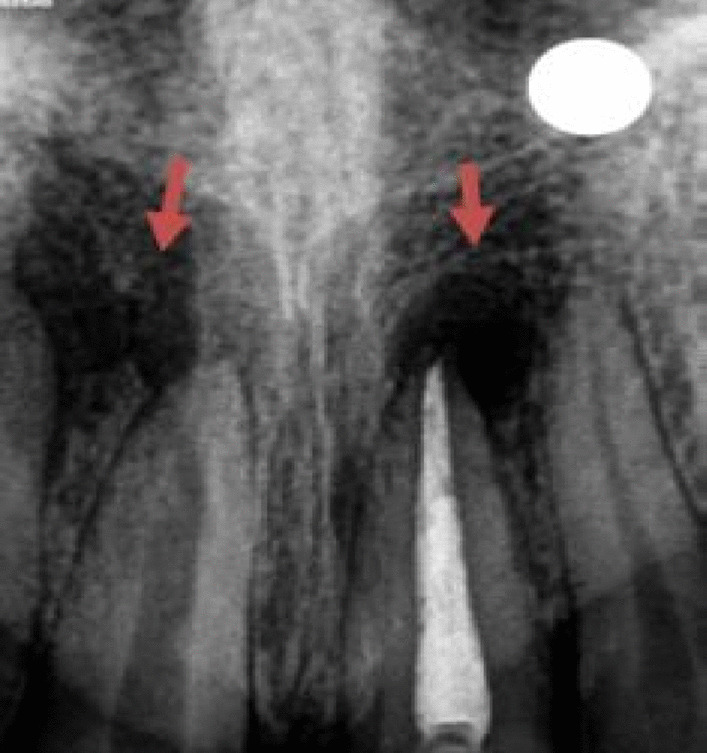
Preoperative periapical radiograph showing 4 to 5 mm of radiolucency around the previously root-treated tooth 21 and 11 with an open apex

Radiographic presentation of the patients included in the study suggested the presence of periapical radiolucency in all patients before the treatment (Fig. 1). The PAI scores correlated to either 4 or 5 in all patients pre-operatively.

After 6 and 12 months of periapical surgery, patients were recalled for the follow-up to evaluate the healing status both clinically and radiographically. The healing group comprised of patients who presented without any complaint of pain, swelling, sinus tract, and/or tenderness to percussion at the end of the follow-up period whereas the non-healing group comprised of patients who presented with any of the above-mentioned signs and symptoms. The clinical follow-up findings of these patients are summarized in Table 3. It was observed that out of 27 patients, 11 patients were classified as non-healers, whereas 16 patients were classified as healers at the end of 6 months. At the end of 12 months, only 6 patients were considered as non-healers, while the rest of the patients were clinically healed.

**Table 3:** Clinical presentation of the study participants after periapical surgery

Clinical parameter	6-Months follow up after periapical surgery			12-Months follow up after periapical surgery		
	Healing status	N	%	Healing status	N	%
Absence of pain/swelling/sinus tract and/or tenderness to percussion	Healing	16	59.25	Healing	21	77.77
Presence of pain/swelling/sinus tract and/or tenderness to percussion	No healing	11	40.74	No healing	6	22.22

Healing and non-healing were also characterized based on radiographic presentation. The periapical index (PAI) was used to assess the outcome of radiographic healing. PAI scores 1 and 2 were regarded as healing, whereas PAI scores 3 to 5 were considered non-healing. The radiographic  findings after the follow-up period are summarized in Table 4. The periapical radiolucency did not increase in any patient after 6 and 12 months. Out of 27, healing was observed in 15 (55.55%) patients after 6 months and 21 (77.77%) patients after 12 months. 12 patients (44.44%) presented with no healing at the end of 6 months and only 6 patients (22.22) reported no healing at the end of 12 months.

**Table 4:** Radiographic evaluation of patients after periapical surgery based on PAI

Outcome	After 6-months		After 12-months	
	Frequency	Percentage	Frequency	Percentage
Healing	15	55.55	21	77.77
No healing	12	44.44	6	22.22

### Histopathological analysis

**Fig. 2:**
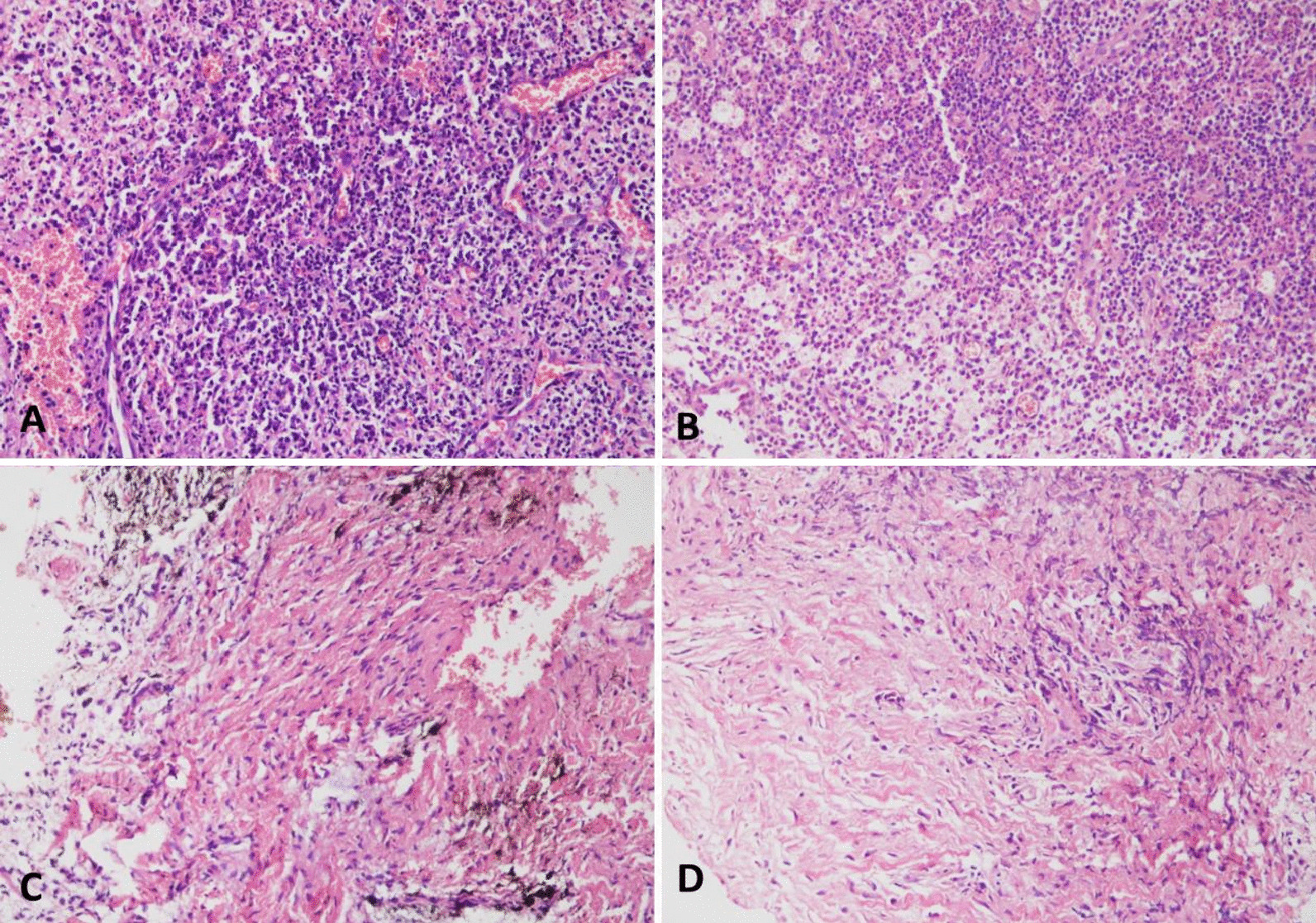
The histopathological images show periapical granuloma in tissues from the healing and non-healing groups: Healing group (**A**–**C**): **A** the granulation tissue with prominent blood vessels showed mixed inflammatory infiltrate comprising of histiocytes, lymphocytes, and plasma cells, can be labeled as an intermediate stage of periapical granuloma. (Hematoxylin and Eosin (H & E); original magnification × 400). **B** The periapical granuloma with intense inflammation (numerous neutrophils). In addition, the granulation tissue with prominent foamy macrophages and blood vessels can be seen, can be labeled as an early stage of periapical granuloma. (H & E; original magnification × 400). **C** The granulation tissue showed mixed inflammatory infiltrate comprising of histiocytes, lymphocytes, and plasma cells. In addition, the brown areas represent hemosiderin pigmented fibrous stroma with prominent fibroblasts are also seen which characterize as a late stage of periapical granuloma. (H & E; original magnification: × 400). **D** The histopathological image of no healing group: Periapical granuloma with fibrous connective tissue with a significant number of fibroblasts and infiltration of inflammatory cells (few plasma cells and histiocytes). (H & E; original magnification: × 400)

The tissues from periapical granuloma were analyzed based on the diagnostic criteria by Omoregie et al. and Neville et al*.* [16, 17]. The histopathological images of the healing group predominantly showed periapical granuloma, characterized by the presence of highly developed granulation tissue, prominent blood vessels, and significant infiltration of inflammatory cells consisting of lymphocytes, plasma cells, and histiocytes (Fig. 2, A-C). The histological slide of the non-healing group also showed the histology of periapical granuloma with an abundance of fibrous connective tissue and a significant number of fibroblasts and scanty infiltration of inflammatory cells, and fewer blood vessels (Fig. 2, D).

### Analysis of baseline MMP expression in healing vs non-healing group

**Fig. 3:**
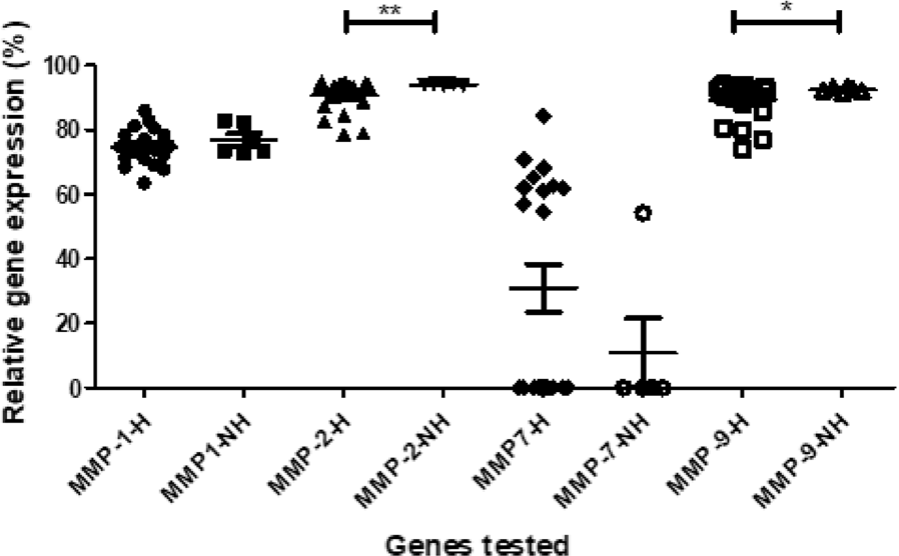
Baseline MMP expression in healing vs non-healing group. The baseline expression of MMPs 1, 2, 7, and 9 was measured in the healing (H) and non-healing (NH) groups. The Y-axis shows the relative expression (%) of each MMP gene tested. The lines with the asterisk sign show a significant difference (*p < 0.05; **p < 0.01) in the expression of MMPs between the healing and non-healing groups

In this study, relative gene expression of MMP1, MMP2, MMP7, and MMP9 was determined at the baseline. We found the mean relative expression of MMP2 and MMP9 to be significantly increased in the non-healing group (94% and 89%, respectively; *p* < 0.05) at the baseline, as compared to the healing group (90.59% and 89%; Fig. 3). Additionally, the mean relative expression of MMP7 at the baseline was less in the non-healing group as compared to the healing group, however, the difference was not statistically significant (Fig. 3).

In the next step, we applied the Pearson correlation test to determine the correlation in the relative expression of the MMP genes in the healing vs non-healing group (Tables 5). In the healing group, a moderate positive correlation was observed between MMP-1, MMP-2, and MMP-9 (*p* < 0.05; Table 5), while a strong positive correlation between MMP-2 and MMP-9 (*p* < 0.05; Table 5). Interestingly, MMP-7 exhibited a weak negative correlation with MMP-2 and MMP-9 (*p* < 0.05; Table 5). In the non-healing group, no statistically significant correlation was observed between any MMPs (Table 5).

**Table 5:** Correlation between MMPs gene expression in the healing and non-healing group

Genes	Healing group			
	MMP-1	MMP-2	MMP7	MMP-9
MMP-1	–	0.59	− 0.18	0.59
MMP-2	0.59	–	− 0.37	0.97
MMP7	− 0.19	− 0.37	–	− 0.42
MMP-9	0.59	0.97	− 0.42	–

Genes	Non-healing group			
	MMP1	MMP-2	MMP-7	MMP-9
MMP1	–	− 0.25	0.57	0.76
MMP-2	− 0.25	–	0.15	0.13
MMP-7	0.57	0.15	–	0.24
MMP-9	0.76	0.13	0.24	–

The table shows the correlation coefficient (r) value between each gene pair, where genes pairs exhibiting statistically significant (*p* < 0.05) correlation are underlined

## Discussion

The current study was conducted to evaluate the baseline expression of MMPs and their relation to periapical wound healing after surgical endodontic treatment. The result of the study showed a statistically significant difference between the mean relative expression of MMP2 and MMP9 in the healing vs non-healing group, and hence, the null hypothesis (that there is no correlation between baseline MMPs 1, 2, 7, 9 expressions in periapical granuloma and periapical wound healing after surgical endodontic treatment) was rejected.

Matrix metalloproteinases (MMP) are an important family of proteolytic enzymes that are associated with inflammation, tissue remodeling, and wound healing [11]. Under normal circumstances, low levels of MMPs are expressed but the level of expression is significantly increased during inflammation and wound healing [11]. In the present study, a statistically significant difference was found in the mean relative expression of MMP2 and MMP9 in the non-healing group (94% and 89%, respectively; *p* < 0.05) at the baseline, as compared to the healing group (90.59% and 89%). The current finding was also supported by Faustino et al. [18], who reported a significantly higher expression of MMP-2 and MMP-9 in symptomatic periapical lesions and suggested their vital role in the etiology and progression of the disease. They further demonstrated higher expression of these enzymes in periapical granulomas as compared to periapical cysts. A recent study by Takahama et al. [19] concluded that periapical granulomas exhibit elevated MMP-2 expression, while decreased expression of OPG, and greater RANKL:OPG ratio in the presence of the endodontic pathogen, Actinobacteria.

In the healing group, we also found a significant positive correlation between the mean expression of MMP1 and MMP2 and MMP9, while a weak negative correlation between MMP-7 and MMP-2 and MMP-9. The correlation between expression of MMP-1, MMP-2, MMP-3, MMP-7, MMP-8, MMP-9, MMP-13, MMP-14, MMP-16, MMP-19, and MMP-25 are reported by numerous researchers in patients with periapical inflammation [13, 20, 21]. However, to the best of our knowledge, none of the researchers have reported the correlation between the baseline expression of matrix metalloproteinase and periapical wound healing after endodontic surgery. Various studies have supported the role of MMPs in wound healing, where MMP1, MMP2, MMP7, and MMP9 have been shown to play a significant part in chronic apical abscesses [9–11]. The findings of the current study were also corroborated by Dezerega et al*.* [22] and Martinho et al*.* [23], who reported the overexpression of MMP-2 and MMP-9 along with pro-oxidative profile in the periapical lesion as compared to healthy tissue. The higher mRNA expression level of MMP-9 might be due to DNA un-methylation of the promoter region when compared with normal healthy tissues. Carneiro et al*.* [24] also confirmed the involvement of MMP-9 along with CD68 + and some inflammatory cells through immune-histochemical staining and real-time PCR, in the degradation of Extracellular Matrix (ECM) in apical periodontitis lesions. Previously, Shi et al*.* [25] showed the down-regulation of MMP-7 via wound healing assay by analyzing the activity of Andrographolide against colorectal carcinoma, but the expression of MMP-2 and MMP-9 remain unchanged. On the contrary, a research study has indicated that the increased levels of MMP-7 play a significant role in the closure of the intestinal epithelial wound and the rate of wound closure was delayed when the activity of MMP-7 was blocked. They also proposed that for normal wound closure, a marginal increase in MMP-7 is crucial; nevertheless, its overexpression might slow down the healing of the intestinal epithelial wound [26]. In a study, researchers investigated the role of serum MMP-2, MMP-3, and MMP-7 in traumatic war wounds and compared it between normal and impaired wound healing. They found out the high serum levels of MMP-7 and MMP-2 and decreased levels of MMP-3 [27].

A 1-year follow-up executed in the current study is a considerably reasonable time for periapical healing evaluation; however, Kruse et al. [18] demonstrated that 1 year is inadequate for successful healing evaluation. They emphasized that different determinants not associated with surgical endodontic treatment may influence the results in the long term. However, Song et al. [19] reported no difference in results between 6 and 10 years follow up time in a prospective study in which the success of 93.3% after microsurgical treatment stayed the same after 5 years. The periapical granuloma can be characterized into three stages, i.e. early stage, intermediate stage, and late-stage based on earlier studies and the diagnostic criteria [16, 17]. The early stage is represented by the presence of granulation tissue with numerous neutrophils, prominent foamy macrophages, and blood vessels, while the intermediate stage constitutes highly developed granulation tissue, prominent blood vessels, and significant infiltration of inflammatory cells consisting of lymphocytes, plasma cells, and histiocytes. Late-stage periapical granuloma exhibited fibrous connective tissue with a significant number of fibroblasts and infiltration of inflammatory cells. Interestingly, late-stage of periapical granuloma was found in the non-healing group, while early and intermediate stages were noticed in the healing group. Surprisingly, this unique correlation of periapical wound healing after surgical endodontic treatment with different stages of periapical granuloma is lacking in the research literature.

It is also worth mentioning that the quality of the surgical endodontic treatment was maintained by using the retrograde filling material with good sealing characteristics such as MTA [28]. Moreover, to avoid the risk of bias, apicoectomy was performed by the principal investigator only, and the radiographic interpretation was carried out by two blinded endodontists. The inter-examiner reliability in evaluating the postoperative radiographs was also calculated. The findings of this study must be seen considering some limitations. First, conventional surgical endodontic treatment was provided to patients due to the unavailability of endodontic microscopes and microsurgical instruments. Second, the study was conducted on single-rooted teeth only. It is also inevitable to mention that the small sample size was taken owing to the availability/consent of the patients, the results might be different for a larger sample therefore results should be interpreted cautiously. Finally, only baseline MMP expression was tested, while MMP expression at 6 and 12 months should have been considered. This objective, however, could not be completed on ethical grounds, and lack of patient consent as the collection of tissue samples months after surgery, especially in healers, would have been an unnecessary surgical procedure. Nonetheless, this study highlights the significance of baseline MMP expression and wound healing. Further studies may suggest MMP as putative prognostic biomarkers for periapical wound healing or non-healing.

## Conclusion

In summary, overexpression of MMP2 and MMP9 may be considered as a potential prognostic biomarker for periapical wound healing after surgical endodontic treatment. Hence, further studies are desirable to establish its precise relationship with periapical wound healing.

## Data Availability

All data generated or analyzed during this study are included in this published article.
